# Different Roles for Tet1 and Tet2 Proteins in Reprogramming-Mediated Erasure of Imprints Induced by EGC Fusion

**DOI:** 10.1016/j.molcel.2013.01.032

**Published:** 2013-03-28

**Authors:** Francesco M. Piccolo, Hakan Bagci, Karen E. Brown, David Landeira, Jorge Soza-Ried, Amelie Feytout, Dylan Mooijman, Petra Hajkova, Harry G. Leitch, Takashi Tada, Skirmantas Kriaucionis, Meelad M. Dawlaty, Rudolf Jaenisch, Matthias Merkenschlager, Amanda G. Fisher

**Affiliations:** 1 Lymphocyte Development Group, MRC Clinical Sciences Centre, Imperial College London, Hammersmith Hospital Campus, Du Cane Road, London W12 0NN, UK; 2 Reprogramming and Chromatin Group, MRC Clinical Sciences Centre, Imperial College London, Hammersmith Hospital Campus, Du Cane Road, London W12 0NN, UK; 3 Wellcome Trust - Medical Research Council Stem Cell Institute, University of Cambridge, Tennis Court Road, Cambridge CB2 1QR, UK; 4 Department of Stem Cell Engineering, Institute for Frontier Medical Sciences, Kyoto University, Kyoto 606-8507, Japan; 5 Ludwig Institute for Cancer Research, Oxford OX3 7DQ, UK; 6 Whitehead Institute for Biomedical Research, MIT, Cambridge, MA 02142, USA

## Abstract

Genomic imprinting directs the allele-specific marking and expression of loci according to their parental origin. Differential DNA methylation at imprinted control regions (ICRs) is established in gametes and, although largely preserved through development, can be experimentally reset by fusing somatic cells with embryonic germ cell (EGC) lines. Here, we show that the Ten-Eleven Translocation proteins Tet1 and Tet2 participate in the efficient erasure of imprints in this model system. The fusion of B cells with EGCs initiates pluripotent reprogramming, in which rapid re-expression of Oct4 is accompanied by an accumulation of 5-hydroxymethylcytosine (5hmC) at several ICRs. Tet2 was required for the efficient reprogramming capacity of EGCs, whereas Tet1 was necessary to induce 5-methylcytosine oxidation specifically at ICRs. These data show that the Tet1 and Tet2 proteins have discrete roles in cell-fusion-mediated pluripotent reprogramming and imprint erasure in somatic cells.

## Graphical abstract

**Graphical Abstract:**

**Figure:**
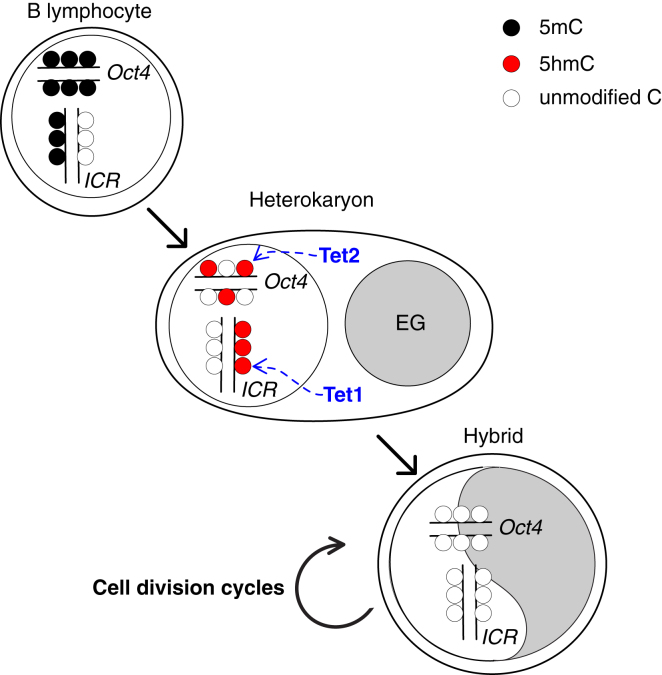

**Highlights:**

► EGCs can erase DNA methylation at ICRs in somatic cells after fusion ► EGCs selectively induce 5hmC accumulation at ICRs in the somatic genome ► Conversion of 5mC to 5hmC at these imprinted domains requires Tet1 ► Tet2 depletion results in delayed reprogramming by EGCs

## Introduction

During mammalian embryogenesis, the genome encounters two waves of global DNA demethylation. The first wave enables the genomes of the contributing gametes to reattain pluripotency, a state that, although transient within the inner cell mass of the mouse blastocyst, is susceptible to in vitro immortalization through the generation of embryonic stem cell (ESC) lines. A second wave of demethylation occurs within primordial germ cells (PGCs), a population that originates from the pluripotent epiblast. Following their specification beginning at embryonic day (E) 7.25 (Ginsburg et al., 1990), PGCs migrate through the dorsal mesentry to the genital ridges (Hayashi and Surani, 2009). Demethylation of imprinted genes occurs after PGCs enter the genital ridge between E11.5 and E13.5 (Hajkova et al., 2002; Hayashi and Surani, 2009). Self-renewing pluripotent embryonic germ cell (EGC) lines can be derived from PGCs from E8.5 onward (Tada et al., 1998; Durcova-Hills et al., 2006; Leitch et al., 2010). Although EGC lines share many features with ESCs (Mise et al., 2008; Hayashi and Surani, 2009; Leitch et al., 2010), they commonly show DNA hypomethylation at imprinted domains, a characteristic that probably reflects their PGC origin (Labosky et al., 1994).

How DNA methylation is reversed is a central question in epigenetic reprogramming (Hayashi and Surani, 2009; Chen and Riggs, 2011). Loss of 5mC from the genome is postulated to occur either through active removal or conversion of 5mC in a manner that does not require DNA synthesis or by passive demethylation, a process in which 5mC or its derivatives are progressively diluted during DNA replication. Among the candidate processes and factors implicated in the active conversion of 5mC to its unmodified form are bifunctional 5mC-specific DNA glycosylases (such as ROS1 and DME) that have been detected in plants (Morales-Ruiz et al., 2006) but not in metazoans. Several enzymes catalyze the deamination or oxidation of 5mC in vertebrates, including members of the AID, APOBEC, and Tet1–Tet3 families, respectively (Muramatsu et al., 2000; Tahiliani et al., 2009; Ito et al., 2010). Thymine DNA glycosylases that excise G-T mismatches or formylcytosine and carboxycytosine from DNA (Ito et al., 2011; Maiti and Drohat, 2011) and initiate the base excision repair pathway (Wu and Zhang, 2010) have also been implicated in DNA methylation loss. Other pathways, including nucleotide excision repair and the associated factor Gadd45a, may also participate in active DNA demethylation (Barreto et al., 2007). From these studies, a wide range of mechanisms for achieving demethylation have been proposed that may operate in vivo (Rai et al., 2008; Guo et al., 2011; Shearstone et al., 2011), in ESCs or during early preimplantation development (Inoue and Zhang, 2011; Williams et al., 2011a; Wu and Zhang, 2011; Xu et al., 2011), within the germline (Hajkova et al., 2010; Popp et al., 2010), and during experimental reprogramming (Bhutani et al., 2010). Despite this, there is no consensus as to whether multiple alternative routes of demethylation act in vivo and in vitro according to context or whether a single universal mechanism predominates (Wu and Zhang, 2010; Teperek-Tkacz et al., 2011).

During cell-fusion-mediated reprogramming, lineage identity is reset and genome methylation is modified (Tada et al., 1997; Pereira et al., 2008; Yamanaka and Blau, 2010). Fusion of differentiated cells, such as lymphocytes or fibroblasts, with mouse ESCs results in heterokaryon (2n + 2n) formation, in which both nuclei are initially discrete. Later, these nuclei fuse and generate tetraploid (4n) hybrids that can proliferate extensively in culture. Upon heterokaryon and hybrid formation, gene expression of the differentiated cell is gradually extinguished in favor of pluripotency (Tada et al., 2001; Pereira et al., 2010; Piccolo et al., 2011). Although ESCs and EGCs can both dominantly reprogram in such assays, EGCs alone have been shown to induce DNA demethylation and erasure of the genomic imprints within the somatic genome upon hybrid formation (Tada et al., 1997). Here, we revisit these pioneering experiments to examine the early molecular events that underlie imprint erasure in somatic cell reprogramming in heterokaryons and hybrids. We show that Tet2 is important for the rapid re-expression of pluripotency-associated genes induced after fusion with EGCs and that it mediates the efficient oxidation of 5mC at the somatic *OCT4* locus. Tet1, in contrast, was required for 5hmC accumulation at ICRs. Our studies reveal key differences in the factors and kinetics regulating the demethylation of the somatic genome and suggest that DNA replication-dependent and -independent mechanisms probably cooperate to erase imprints in this model system.

## Results

### Loss of DNA Methylation at ICRs Is Induced in Somatic Cell Hybrids Generated with EGCs

**Figure 1:**
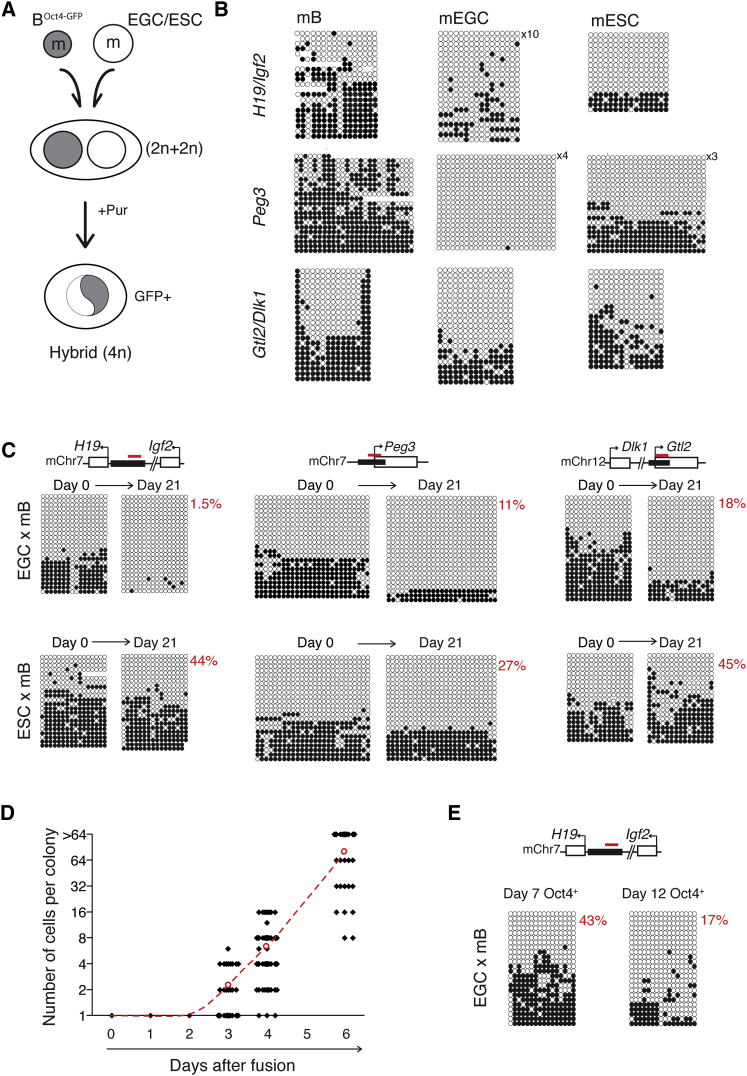
Reprogrammed Hybrids Generated with Mouse EGCs Show Reduced DNA Methylation at Imprinted Loci (A) Oct4-GFP re-expressing (GFP^+^) hybrids were generated by fusing puromycin-resistant (Pur^R^) mouse B^*Oct4*-*GFP*^ cells (gray) with mouse EGCs or ESCs (white). (B) Bisulfite analysis of *H19*/*Igf2*, *Peg3*, and *Gtl2*/*Dlk1* loci in parental cells before fusion where unmethylated and methylated CpG are shown as open and closed circles, respectively. (C) Bisulfite analysis of cells before fusion (day 0) and after hybrid formation (day 21). Hybrids generated with ESCs were used as controls. ICR positions are marked in black, red bars indicate the primer-amplified PCR products, and percentage methylation levels are shown in red. (D) Number of cells in hybrid colonies generated by fusing mB and mEGCs. Values for individual colonies (black squares) and average cell number (red open circles) are shown at each time point. (E) Bisulfite analysis of *H19*/*Igf2* locus in samples 7 and 12 days after fusion of mEGCs and mB^*Oct4*-*GFP*^ cells. See also Figure S1.

Previous studies have shown that fusion of mouse thymocytes with EGC lines 58G and 55G generated hybrids, in which the T cell genome was hypomethylated at ICRs and a model imprinted transgene was reactivated (Tada et al., 1997). To verify and extend these studies, we performed sodium bisulfite analysis to quantify DNA methylation at the mouse *H19*/*Igf2*, *Peg3*, and *Dlk1*/*Gtl2* ICRs in EGCs, in a puromycin-resistant mouse B line that carries a silent *Oct4*-*GFP* transgene (mB), and in reprogrammed hybrids isolated 21 days after fusion between these cell types on the basis of puromycin resistance and Oct4-GFP re-expression (GFP^+^) (Figure 1A, and Figure S1A available online). Hybrid clones were also generated with the use of ESCs for comparison, and both sets of hybrids were checked for assessment of karyotypic fidelity. Bisulfite analysis confirmed that several imprinted domains were hypomethylated in EGCs (<1% *Peg3*, 13% *H19*/*Igf2*, and 24% *Dlk1*/*Gtl2*) in comparison to mB, in which approximately half of alleles were methylated (50%–61%, Figure 1B). Hybrids formed with EGCs showed a reduction in DNA methylation at *H19*/*Igf2* (from 30% of alleles at day 0 to 1.5% by day 21), *Peg3* (from 34% to 11%), and *Dlk1*/*Gtl2* (43% to 18%) (Figure 1C, upper panels). Hybrids generated with ESCs did not show substantive changes in DNA methylation at any of these loci (Figure 1C, lower panels). The selective loss of 5mC at ICRs in EGC-derived hybrids was confirmed by HpaII restriction digests (Figure S1B) and was verified with the use of multiple independently isolated hybrid clones (Figure S1C). In addition, B cell hybrids generated after fusing mB cells with female Pgk12 ESCs (Zvetkova et al., 2005), or ESCs lacking *Dnmt1* (Li et al., 1992) or *Dnmt3a* and *Dnmt3b* (Chen et al., 2003), did not show comparable reductions in methylation at imprinted regions, despite each of the parental ESC lines being hypomethylated (Figures S1D and S1E). These data show that EGCs induce DNA demethylation at imprinting domains within the somatic genome upon cell-fusion-mediated reprogramming, whereas ESC lines with a similarly low level of endogenous DNA methylation do not.

To investigate the kinetics of this loss of DNA methylation, we fused transgenic *Oct4*-*GFP* mB cells with EGCs and plated them at low density so that the timing of Oct4-GFP re-expression and cell division could be assessed with microscopy. During the first couple of days after fusion, individual heterokaryons were detected. Thereafter, cells re-expressing Oct4-GFP were observed and hybrid colonies containing increasing numbers of cells were recorded at successive days (Figure 1D) so that, by days 6–7, most clones had undergone at least five rounds of cell division. Bisulfite analysis of reprogrammed (Oct4-GFP^+^) hybrid clones isolated at this stage showed substantial DNA methylation remaining at the *H19*/*Igf2* locus (43%, day 7) that declined as these clones were propagated further (17%, day 12; <5%, day 21) (Figures 1E and S1C). These results suggested that EGC-induced DNA demethylation at imprinted regions might be slower or less efficient than EGC-induced demethylation and reactivation of the *Oct4* locus.

### Demethylation and Re-expression of Maternal Peg1

**Figure 2:**
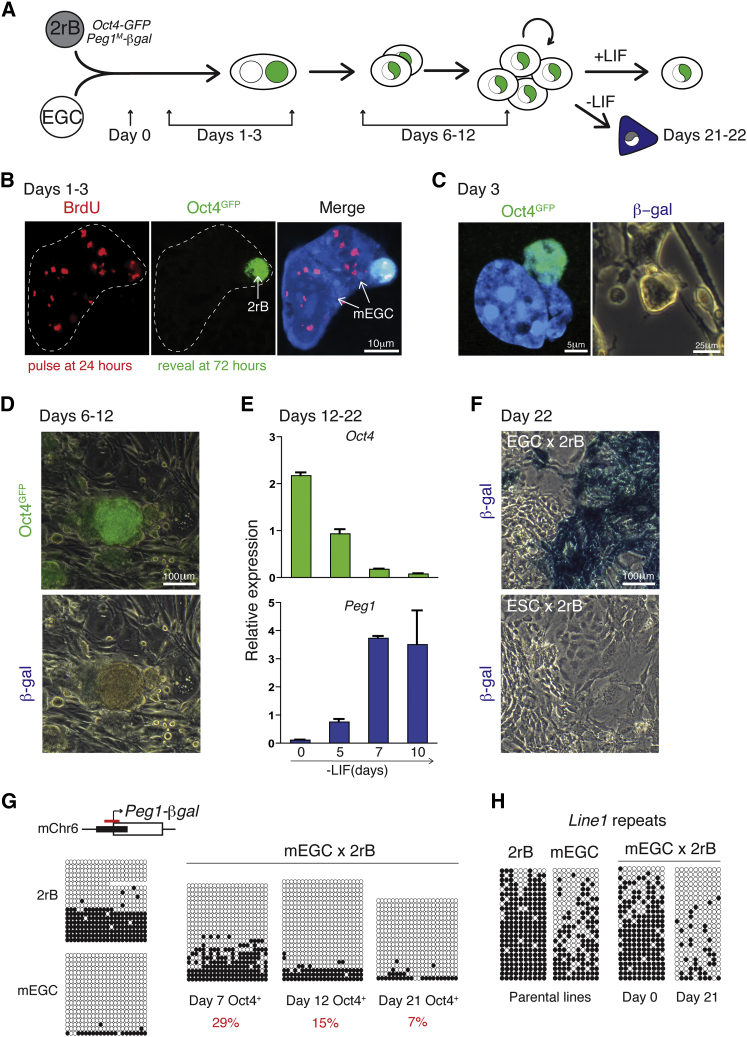
Early Pluripotent Reprogramming and Late Re-Expression of an Imprinted *Peg1* Allele in Mouse B Cells After Fusion with EGCs (A) Reprogramming and imprint erasure was modeled with the use of mouse 2rB cells (puromycin-resistant *Oct4*-*GFP* B cells containing a maternal *LacZ* knock-in allele of *Peg1* [*Peg1*^*M*^-*βgal*]). (B) A heterokaryon between EGCs and 2rB cells showing BrdU incorporation (pulse-labeled at day 1, red), Oct4 re-expression (Oct4-GFP^+^ day 3, green), and DAPI staining (blue). (C) A representative Oct4-GFP^+^ (green) EGC-2rB heterokaryon is shown in which β-galactosidase activity was not detected at day 3. (D) Hybrid colonies at day 12 remain Oct4-GFP^+^ but lack β-galactosidase activity. (E) Kinetic analysis of *mOct4* (green) and *mPeg1* (blue) gene expression in 2rB × mEGC hybrids following LIF withdrawal. Mean and SD of three independent experiments are shown, and values were calculated relative to *UBC*. (F) Maternally derived *Peg1*-βgal activity (blue) is selectively detected in 2rB × EGC hybrids after differentiation induced by LIF withdrawal. (G) Bisulfite analysis showing the kinetics of DNA methylation loss at *Peg1* in 2rB cells following fusion with EGCs. Parental cells before fusion (left panel) and in Oct4-GFP^+^ 2rB × mEGC hybrids collected 7, 12, and 21 days after fusion (right panel). Unmethylated (closed circles) and methylated (open circles) CpGs are indicated and the percentage methylation is shown in red. Red bars indicate the position of primer-amplified PCR product relative to the *Peg1*-*βgal* locus. (H) Bisulfite analysis of DNA methylation at *Line1* repetitive elements in parental 2rB and mEGCs before and after hybrid formation. See also Figure S2.

To investigate this further, we established a puromycin-resistant, dual-reported mouse B cell line (2rB) that carried a maternal *LacZ* knockin allele of *Peg1* (Lefebvre et al., 1998) in addition to the *Oct4*-*GFP* transgene (Yeom et al., 1996). This allowed for the coordinate monitoring of events associated with pluripotent reprogramming (Oct4-GFP expression) and *Peg1*-*LacZ* reactivation (β-galactosidase activity), as depicted in Figure 2A. Mouse 2rB cells were fused with EGCs in a 1:1 ratio and plated at low density. Oct4-GFP was detected from day 3 onward in heterokaryons (Figure S2A) as well as in newly formed two-cell hybrids. Given that DNA synthesis by somatic nuclei is induced after fusion with ESCs (Tsubouchi et al., 2013), we asked whether 2rB cells undergo DNA synthesis when fused with EGCs. For this, heterokaryons were pulsed with BrdU (45 min, 100 μM) 24 hr after fusion and cultured for an additional 2 days before BrdU incorporation was revealed among the reprogrammed (Oct4-GFP^+^) cells. We found that most GFP^+^ heterokaryons identified at day 3 (>75%) had incorporated a BrdU pulse applied 24 hr after fusion (illustrated in Figure 2B), similar to results obtained with ESCs (Tsubouchi et al., 2013). *Peg1*-regulated β-galactosidase activity was not detected in any cells 3, 6, or 12 days after fusion (Figures 2C and 2D), consistent with the reported low level of expression of Peg1 in pluripotent stem cells (Lefebvre et al., 1998). *Peg1*^*M*^-*βgal* expression was, however, evident in hybrid cells that had been induced to differentiate by leukemia inhibitory factor (LIF) withdrawal (Figures 2E and 2F) and, importantly, was uniquely detected in 2rB hybrids generated with EGCs, rather than parallel cultures derived from ESCs (Figure 2F). These data demonstrated that EGCs were able to functionally reset the maternally derived (silent) *Peg1* imprint in the B cell genome.

To investigate the kinetics of this reversal, we examined DNA methylation at the *Peg1*-*LacZ* locus among Oct4-GFP^+^ hybrid cells that were harvested at sequential times after fusion. As shown in Figure 2G, bisulfite analysis revealed that the *Peg1* marker locus remained substantially methylated in hybrid cells isolated at day 7 (29%) but declined as these cells were further cultured (15% at day 12, 7% at day 21). Loss of methylation at the *Peg1*-*LacZ* locus in EGC-derived hybrids was confirmed with HpaII digests (Figure S2B). Altogether, these results suggest that demethylation of somatic *Peg1* was induced much more slowly than demethylation occurring at the *Oct4* locus.

To address whether EGC-induced reprogramming erased DNA methylation at additional sites in the somatic genome, we examined the methylation status of long interspersed element (LINE) repeats in reprogrammed 2rB hybrids generated with either EGCs or ESCs. As shown in Figures 2H and S2C, *Line1* repeats were hypomethylated in 2rB hybrids generated with EGCs but remained partially methylated in hybrids formed with ESCs. This suggests that the potent capacity of EGCs to induce demethylation was not restricted to pluripotency-associated genes and ICRs but also extended to other sites of DNA methylation within the somatic genome.

Given that DNA hypomethylation could result from failures in maintaining DNA methylation during cell division, we compared the expression of the DNA maintenance methyltransferase Dnmt1, the de novo methyltransferases Dnmt3a and Dnmt3b, and the tethering factor Uhrf1 in the EGC lines 55G and 58G and in ESL21 ESCs. Gene expression and western blotting analysis confirmed that these factors were present in both EGCs and ESCs (Figures S3A and S3B, respectively). These cell lines also expressed comparable levels of pluripotency-associated genes (Figure S3C). Fluorescence microscopy confirmed a similar distribution of Dnmt1 and Uhrf1 proteins within mouse EGCs and ESCs (Figures S3D and S3E, respectively). We noted a slight trend for increasing ICR hypomethylation with EGC passage (Figure S3F), suggesting that cell division in culture might either directly promote a loss of DNA methylation or select for cells with increased hypomethylation.

### Altered 5mC Levels at Imprinted Domains in EGC Heterokaryons

**Figure 3:**
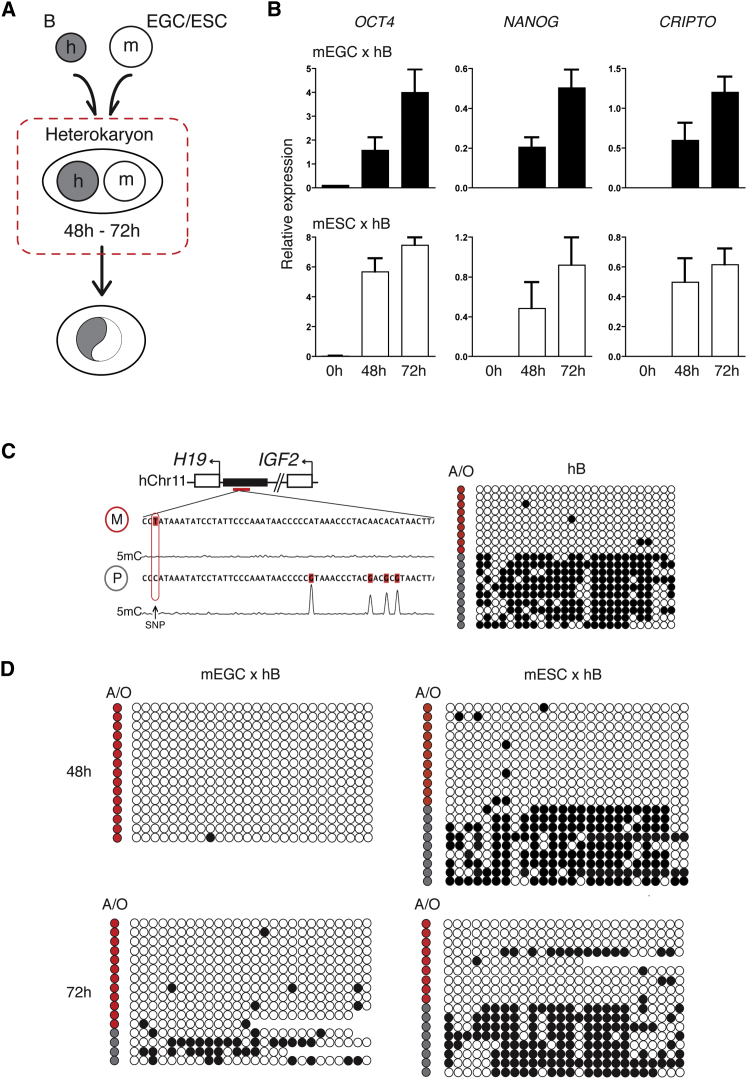
Detection of Paternally Derived *H19*/*IGF2* Alleles by Bisulfite Analysis Is Selectively Compromised in EGC-Induced Heterokaryon (A) Transient heterokaryons formed between human B cells (gray) and mouse EGCs or ESCs (white) were generated 48–72 hr after fusion (dashed red box). (B) Quantitative RT-PCR (qRT-PCR) analysis of human *OCT4*, *NANOG*, and *CRIPTO* expression by human B-lymphocytes before (0 hr), and 48 hr and 72 hr after fusion with either EGCs (black histograms) or ESCs (white histograms). Results are the mean and SE of five independent experiments where values were normalized to *GADPH*. (C) Sequencing analysis of human *H19*/*IGF2* in bisulfite converted DNA isolated from a human B cell clone (hB). Maternally (M, red circle) and paternally (P, gray circle) derived alleles are distinguished by a thymidine-adenine single nucleotide polymorphism (SNP). Bisulfite analysis of hB samples where open and closed circles represent unmethylated and methylated CpGs, respectively, and the allelic origin (A/O) of *H19*/*IGF2* alleles is indicated in red (maternal) or gray (paternal). (D) Bisulfite analysis of *H19*/*IGF2* methylation in hB heterokaryons formed with mEGCs (left) or mESCs (right). See also Figure S3.

To investigate how EGCs induced the remodeling of a somatic genome during reprogramming, we generated interspecies heterokaryons using human B cells. This established approach uses human-specific primer sets, antibodies, and probes in order to discriminate the earliest events that occur in the somatic (human) nucleus after cell fusion (Pereira et al., 2008) (Figure 3A). Fusion of human B cells (hB) with mouse EGCs (1:1) induced the expression of human pluripotent gene transcripts (*OCT4*, *NANOG*, and *CRIPTO*) at comparable levels and with similar kinetics as those in fusions generated with mouse ESCs (Figure 3B). Before evaluating DNA methylation at imprinted domains, we began by sequencing the human *H19*/*IGF2* domain in our human B cell clone and identified a SNP within the ICR that could discriminate the 5mC-containing (paternal) allele from the unmodified maternal allele (Figure 3C). Bisulfite analysis confirmed that methylated *H19*/*IGF2* DNA accounted for approximately 40% of the hB sample before fusion and originated predominantly from the paternal allele. In samples 48 and 72 hr after fusion, most *H19*/*IGF2* DNA sequences detected after bisulfite conversion were apparently unmethylated (99% and 90%, respectively; Figure 3D, left panels). SNP analysis revealed that this surprising result reflected a strong preference in the detection of the unmodified maternal allele rather than bona fide changes to the methylated paternal allele (Figure 3D, allelic origin [A/O]). Importantly, in parallel fusions performed with hB and ESCs (Figure 3D, right panels), this bias was not seen: methylated *H19*/*IGF2* alleles accounted for 42% of the bisulfite converted hB sample before fusion and 38% and 42% at 48 and 72 hr after heterokaryon formation, respectively. Biased detection of maternally derived *H19*/*IGF2* alleles was therefore selective for reprogramming induced by EGCs.

### Evidence that 5mC Is Rapidly Converted to 5hmC at Several ICRs in the Somatic Genome after Fusion with EGCs

**Figure 4:**
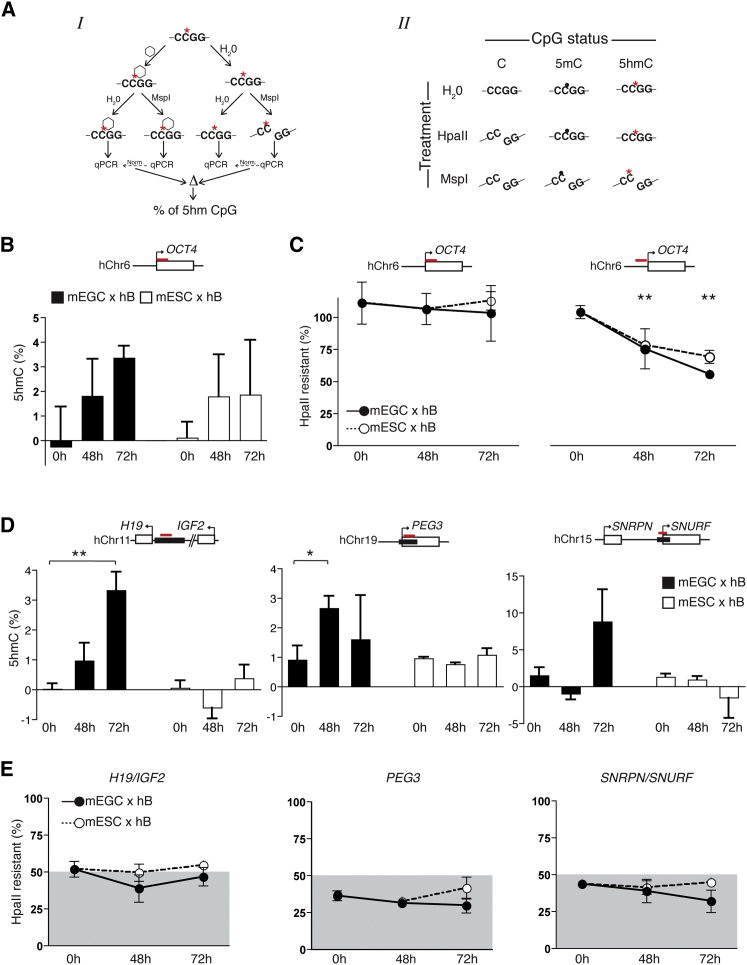
Evidence that 5hmC Levels Increase at ICRs in Somatic Cells after Fusion with EGCs (A) Detection of 5hmC (*I*) and unmodified cytosine (*II*) in human heterokaryon samples. Genomic DNA was divided and was either treated with T4-β-glucosyltransferase, which binds glucose groups selectively at 5hmC sites (red asterisk) and creates 5hgmC (open hexagon, left), or left untreated (H_2_O). Samples were digested with MspI (which does not digest 5hgmC) or left undigested (H_2_O), and the abundance of locus-specific DNA in each was compared by qPCR. In strategy *II*, unmodified (C) and modified CpG (5mC and 5hmC) levels were evaluated by HpaII digestion (right); DNA samples were treated with HpaII (which does not cut 5mC and 5hmC), left undigested (H_2_O), or treated with MspI (which cuts both and provides a positive control). The abundance of locus-specific DNA within each of these samples was estimated by qPCR and used to calculate the percentage of HpaII resistance. (B) Levels of 5hmC at *OCT4* in hB cells before (0 hr), and 48 hr and 72 hr after fusion with mouse EGCs (black bars) or ESCs (white bars) are shown as the mean and SE of three to five independent experiments. (C) HpaII digestion analysis of *OCT4* in hB lymphocytes before (0 hr) and 48 hr and 72 hr after fusion with EGCs (closed circles) or ESCs (open circles) are shown. Red bars mark the position of primer-amplified PCR products derived from the promoter (right) and downstream of the TSS (left), and values represent the mean and SE of three to five independent experiments. ^∗∗^, p value < 0.005 calculated with Student’s t test. (D) Detection of 5hmC at *H19*/*IGF2*, *PEG3*, and *SNRPN*/*SNURF* was evaluated in genomic DNA from human B cells before (0 hr), and 48 or 72 hr after fusion with EGCs (black histograms) or ESCs (white histograms). The position of ICRs is indicated in black, and red bars mark the position of primer-amplified PCR product. The values shown are the mean and SE of three to five independent experiments. ^∗^, p value < 0.05; ^∗∗^, p value < 0.005 calculated with Student’s t test. (E) HpaII digests of *H19*/*IGF2*, *PEG3*, and *SNRPN*/*SNURF* ICRs in hB lymphocytes before (0 hr), and 48 and 72 hr after fusion with EGCs (closed circles) or ESCs (open circles). Anticipated 50% levels of HpaII resistance are marked in gray, and the values shown are the mean and SE of three to five independent experiments.

Impaired detection of methylated, paternally derived human *H19*/*IGF2* alleles in bisulfite assays of EGC-heterokaryons was consistent in repeat experiments (not shown). We reasoned that this could reflect an accumulation of 5hmC at the *H19*/*IGF2* locus given that previous studies had shown that 5hmC (but not 5mC) reacts with sodium bisulfite to yield cytosine 5-methylenesulfonate (CMS) that can stall DNA polymerase and result in inefficient amplification by Taq polymerase (Huang et al., 2010). To explore this possibility, we used two different restriction enzyme digest protocols to compare levels of 5hmC and unmodified cytosine at *OCT4* and at several human ICRs before and after heterokaryon formation with either EGCs or ESCs. In the first approach, pretreatment of samples with T4 glucosyltransferase (which selectively protects 5hmC-containing DNA template from MspI digestion) was used to estimate 5hmC abundance (Figure 4A, left panel *I*). Using this strategy, we detected increased MspI resistance at the human *OCT4* locus (Figure 4B) and at each of the human ICRs examined (*H19*/*IGF2*, *SNRPN*/*SNURF*, and *PEG3*; Figure 4D) in samples harvested 48 and 72 hr after fusion with EGCs (black histograms). This increase in 5hmC at human ICRs was not evident in heterokaryons formed with ESCs (Figures 4D, white histograms), although increased MspI resistance consistent with remodeling of this locus in ESC-mediated pluripotent reprogramming was seen at *OCT4* (Figure 4B, white histograms). These results, along with the bisulfite results shown previously, confirmed that EGCs selectively induced the accumulation of 5hmC at imprinted domains within the somatic genome.

A second enzymatic approach was used to estimate the abundance of unmethylated cytosine at sites in the human genome before and after fusion. This relies on template digestion with HpaII, an enzyme that exclusively cuts unmodified cytosine (Figure 4A, right panel *II*). Using this strategy, we observed only modest increases in the level of unmodified cytosine detected at human ICRs (*H19*/*IGF2*, *SNRPN*/*SNURF*, and *PEG3*) that occurred exclusively in EGC-derived heterokaryons (Figure 4E). However, substantial increases in HpaII sensitivity were detected at the human *OCT4* promoter in both ESC- and EGC-derived samples (Figure 4C, right panel), consistent with *Oct4* demethylation being critical for successful reprogramming to pluripotency (Simonsson and Gurdon, 2004).

### Independent Roles of Tet1 and Tet2 Hydroxylases in Pluripotent Reprogramming and Imprint Erasure

**Figure 5:**
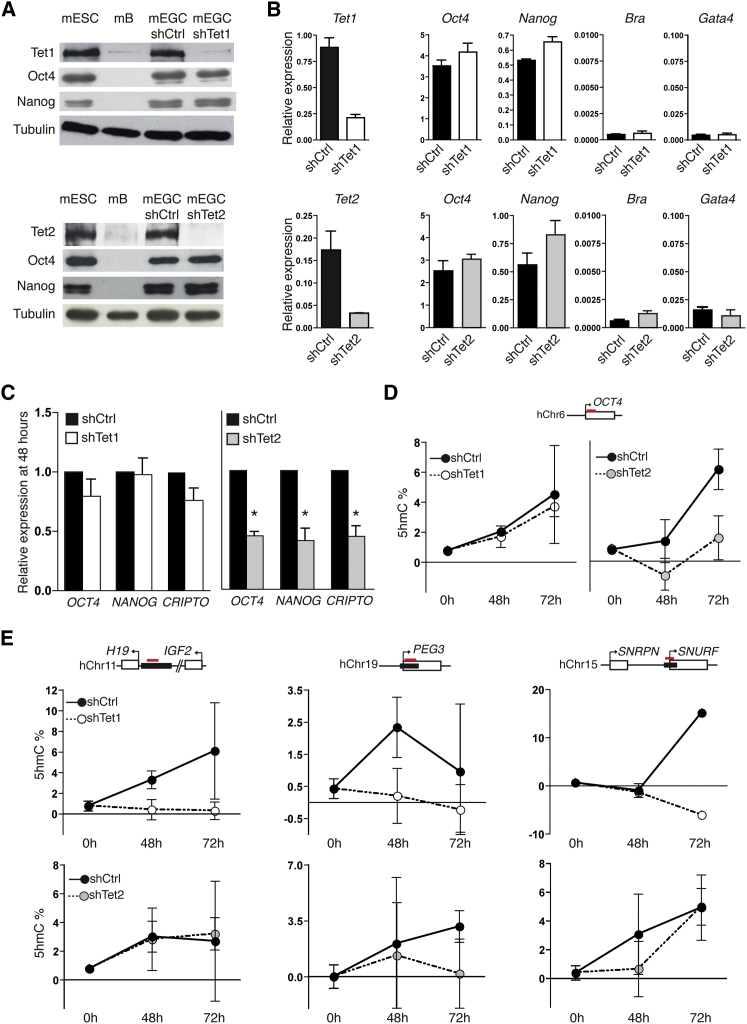
Distinct Roles of Tet Proteins in EGC-Mediated Heterokaryon Reprogramming (A) Western blot detection of Tet1 (upper), Tet2 (lower), Oct4, and Nanog proteins in whole-cell extracts of mouse ESCs (mESCs), mouse B cells (mB), and mouse EGCs after transfection with shRNA for Tet1 (shTet1), for Tet2 (shTet2), or for control vector (shCtrl). Antibodies specific for Tet1, Oct4, Nanog, and Tubulin (a loading control) were used as described in the Supplemental Experimental Procedures. (B) qRT-PCR analysis of *Tet1*, *Tet2*, *Oct4*, *Nanog*, *Brachyury (Bra)*, and *Gata4* expression in mouse EGCs after transfection with shTet1 (white), shTet2 (gray), or control vector (black) where results are the mean and SD of four to five independent experiments and the values were calculated relative to *UBC*. (C) qRT-PCR analysis of *OCT4*, *NANOG*, and *CRIPTO* transcripts detected in hB cells 48 hr after fusion with mouse EGCs transfected with control vector (black), shTet1 (white), or shTet2 (gray). The values shown are expressed relative to human *GAPDH*, normalized to shCtrl, and show the mean and SD of four to five independent experiments. ^∗^, p value < 0.05 calculated with student t test. (D) Levels of 5hmC, estimated using the strategy depicted in *I*, at the human *OCT4* locus in human B cells before (0 hr) and 48 hr and 72 hr after fusion with EGCs previously transfected with shCtrl (closed circles), shTet1 (open circles), or shTet2 (gray circles). Results are the mean and SE of four to five independent experiments. (E) Levels of 5hmC at *H19*/*IGF2*, *PEG3*, and *SNRPN*/*SNURF* ICRs as detailed above in (D). See also Figure S4.

The mammalian TET family comprises three members that share significant sequence homology (Münzel et al., 2011) and a capacity to regulate 5hmC levels (Tahiliani et al., 2009). Tet1 and Tet2 proteins are expressed by pluripotent ESCs (Ito et al., 2010), regulated by Oct4 (Koh et al., 2011), and have been implicated in DNA demethylation during PGC development (Hackett et al., 2013). Tet3 is expressed in the preimplantation zygote, where it has been proposed to mediate the conversion of 5mC to 5hmC immediately after fertilization (Gu et al., 2011; Wossidlo et al., 2011). To investigate whether Tet1 and Tet2 enzymes were important for imprint erasure in our model system, we initially verified their expression in EGC lines. Mouse EGC lines expressed Tet1 and Tet2 proteins at levels that were comparable with ESCs, whereas differentiated controls such as mouse B cells (mB) did not (Figure 5A). RNAi-mediated depletion of Tet1 or Tet2 (shTet1 and shTet2, respectively) in EGCs resulted in the efficient and selective knockdown of Tet1 and Tet2 transcripts and proteins (Figures 5A and 5B) without provoking cell differentiation, as indicated by the sustained high level expression of *Oct4* and *Nanog* and a lack of expression of differentiation-associated genes such as *Bra* and *Gata4* (Figure 5B).

To investigate the contribution of Tet1 and Tet2 hydroxylases in EGC-mediated reprogramming and imprint erasure, we examined the capacity of Tet-depleted EGCs to reprogram human B cells after cell fusion. Knockdown of Tet2 in EGCs consistently reduced their reprogramming efficiency by about 50%, whereas Tet1 depletion had only a mild effect on human B cell reprogramming, as judged by the induction of a panel of human pluripotency genes (Figure 5C). Consistent with this, Tet1 depletion did not significantly impair the accumulation of 5hmC at the *OCT4* locus, whereas Tet2 depletion resulted in a marked decrease in 5hmC levels (Figure 5D) and delayed loss of DNA methylation at the somatic *OCT4* promoter (Figure S4A, left panel). These results allowed us to examine whether Tet1 was required for the conversion of 5mC to 5hmC at human imprinted domains in heterokaryons, independent of a requirement for this protein in pluripotent reprogramming. Although EGCs depleted of Tet1 were able to induce reprogramming of human B cells, they failed to induce 5hmC conversion at *H19*/*IGF2*, *SNRPN*/*SNURF*, and *PEG3* ICRs, as judged by MspI protection (Figure 5E, upper panels). In addition, the depletion of Tet1 completely restored the equivalence of detection of maternal and paternal human *H19*/*IGF2* alleles after bisulfite conversion (Figure S4B), directly implicating Tet1 in the biased amplification of paternal alleles that was detected previously. Depletion of Tet2, in contrast, had a mild effect on 5hmC accumulation at human ICRs (Figure 5E, lower panels), which probably reflects the diminished capacity of these cells to reprogram (Figure 5C). The levels of 5mC detected at ICRs in heterokaryons formed with Tet-depleted EGCs remained relatively constant over the first few days (Figure S4A, right panel), consistent with previous results showing that demethylation occurs later at these domains than at *OCT4* and follows multiple rounds of cell division (Figures 1D and 1E).

Given that DNA synthesis is an early event in both ESC and EGC fusion-based reprogramming (Tsubouchi et al., 2013) (Figure 2B), we asked whether 5hmC accumulation at ICRs required DNA synthesis. To address this, we fused hB cells with mEGCs and treated the resulting heterokaryons with inhibitors of DNA synthesis (aphidicolin or mimosine) for 60 hr before assessing their impact on the induction of 5hmC relative to untreated controls. As shown in Figure S4C, treatment with either drug did not prevent 5hmC accumulation at either of the human ICRs tested (*H19*/*IGF2* and *PEG3*) but comprehensively blocked the induction of human pluripotency-associated genes (Figure S4D). This result is consistent with DNA synthesis being required for pluripotent reprogramming (Tsubouchi et al., 2013), but it also suggests that the initial conversion of 5mC to 5hmC at human imprinted domains occurs through a DNA replication-independent mechanism.

## Discussion

Most imprinted domains contain a gametic differentially methylated region that acquires DNA methylation from the maternal germline in the oocyte. At least four domains, including *H19* and *Dlk1*, are, however, known to carry a paternal imprint that is acquired in the male germline before birth (reviewed by John and Lefebvre, 2011). Here, we show that EGC lines can reset both maternal (*Peg1*, *Peg3*, and *SNRPN*) and paternal (*H19* and *Dlk1)* imprints in B cells upon cellular fusion and provide fresh evidence that this is initiated as heterokaryons are formed. Not all mouse EGC lines share this property, and it is noteworthy that the female and male EGC clones used here (58G and 55G, respectively) were both isolated late during PGC development (E12.5) and require a low passage number and specialized culture conditions to retain their capacity to erase imprints after fusion. In practice, we have found that these cell lines begin to lose their ability to erase imprints in somatic cells by passage 30, despite a trend for these EGCs to become increasingly hypomethylated in culture (Figure S3F). Female ESCs and ESCs that lacked Dnmt1 or Dnmt3a and Dnmt3b also showed global hypomethylation but were consistently unable to erase methylation at ICRs in somatic partners. This suggests that EGCs isolated from PGCs at particular stages in development are uniquely able to target and impose demethylation at imprinted domains within the somatic genome. Because ESCs and EGCs express similar levels of Tet1, Tet2, Dnmt3a, and Dnmt3b proteins and show similar distributions of Dnmt1 and Uhrf1, the molecular details of this selective demethylation are not fully understood.

Recent time-course studies of developing mouse PGCs have suggested that the progressive loss of DNA methylation in cells from E8 onward occurs in discrete temporal phases in which different genic and intergenic regions are affected (Seki et al., 2005; Guibert et al., 2012). A loss of methylation at germline-specific genes and some somatic genes is detected relatively early (E8–E9), whereas imprinted domains such as *Peg3*, *H19*/*Igf2*, and *Line1* elements are targeted later (E9.5–E12.5) and mobile elements including *intracisternal A*-*particles* and *LTR*-*ERV1* retroelements remain relatively resistant to erasure (Hajkova et al., 2002; Guibert et al., 2012). On the basis of kinetic observations, it was originally suggested that replication-dependent demethylation might operate early in PGC development, whereas replication-independent mechanisms function later as PGCs enter the gonads and G2 phase cells accumulate. However, recent studies of DNA methylation dynamics (Hackett et al., 2013; Seisenberger et al., 2012) have suggested that high levels of Tet1 and Tet2 expression at E9.5–E10.5 could drive conversion of 5mC to 5hmC at ICRs and that reprogramming and imprint erasure may then occur by “replication-coupled” dilution (Kagiwada et al., 2013). Evidence linking putative hydroxylase and deaminase family members with DNA demethylation in vivo (Popp et al., 2010; Gu et al., 2011; Inoue and Zhang, 2011; Iqbal et al., 2011) as well as in vitro (Rai et al., 2008; Bhutani et al., 2010) has been widely reported. Mice that are deficient in AID, Tet1, or Tet2 are, however, viable and largely fertile, although Tet1-null females were recently shown to have reduced numbers of germ cells and to fail to properly reactivate meiotic genes (Yamaguchi et al., 2012). This suggests that, although factors such as Tet1, Tet2, and AID might be nonessential or redundant during PGC development in vivo (Muramatsu et al., 2000; Dawlaty et al., 2011; Li et al., 2011), these factors may be indispensable for ICR remodeling in stringent in vitro assays. Consistent with this idea, the maternally methylated *Peg1* and *Peg3* ICRs were found to show similar hypomethylated profiles in mature sperm DNA obtained ex vivo from *Tet1*-null, heterozygous, and matched wild-type control mice (Figure S4E).

The role of active (replication-independent) and passive (replication-dependent) DNA demethylation in global reprogramming has been widely contested. Initial claims that heterokaryon-mediated reprogramming by ESCs requires AID and occurs independently of DNA synthesis (Bhutani et al., 2010) have been called into question by recent studies (Foshay et al., 2012; Tsubouchi et al., 2013). Induced pluripotent stem cell (iPSC)-based investigations have shown that reprogramming is enhanced by cell division (Hanna et al., 2009) and have indicated that Tet2 and Parp1 are required to direct 5hmC accumulation and subsequent transcriptional induction of pluripotency-associated genes (Doege et al., 2012). The in vitro reprogramming studies presented here show that depletion of Tet1 or Tet2 reduces the efficiency of EGC-induced reprogramming of somatic cells. We identify two early steps in imprint erasure that are required to induce efficient locus remodeling and gene re-expression: precocious DNA synthesis and Tet1-mediated conversion of 5mC to 5hmC at ICRs. Given that drugs that block DNA polymerase activity prevented pluripotent gene re-expression but only minimally affected 5hmC accumulation at ICRs, our study suggests that both active and passive mechanisms are required for efficient reprogramming and imprint erasure.

We have shown that demethylation of pluripotency genes (such as *Oct4*) occurs relatively early after cell fusion and relies on Tet2. This is consistent with a recent report that shows that Tet2 is critical for iPSC-based reprogramming (Doege et al., 2012). On the other hand, our data indicate that the demethylation of ICRs in our reprogramming model depends on Tet1. Although detection of 5hmC at ICRs was initiated early after EGC fusion and did not require DNA polymerase activity, the acquisition of a hypomethylation state became evident only at later stages (days 7–12) after cells had undergone several rounds of cell division. These data suggest that Tet1 and Tet2 target different sites in the somatic genome (possibly through the Tet1 CXXC domain) (Tahiliani et al., 2009), although they do not exclude that somatic loci have variable thresholds of sensitivity to Tet-based remodeling. Given that 5hmC is not recognized by the maintenance methyltransferase Dnmt1 (Hashimoto et al., 2012), conversion of 5mC to 5hmC at specific sites in the somatic genome would be predicted to promote passive demethylation as hybrid cells divide. Thus, a two-step model for ICR remodeling could provide an explanation for the delay between 5hmC detection (at day 2) and locus demethylation (at days 7–12) seen at mouse *Peg1* and other ICRs studied herein. In this model, active (mediated by Tet1) and passive (mediated through DNA synthesis and cell division) mechanisms could contribute to imprint erasure in vitro.

Our study shows that Tet1 and Tet2 proteins have distinct roles in pluripotent reprogramming induced by EGCs. Others have shown that prolonged depletion of Tet1 skews ESC differentiation toward trophectoderm and mesendoderm (Koh et al., 2011), whereas Tet2 depletion skews hematopoietic differentiation (Ko et al., 2011) and impairs the *trans*-differentiation of pre-B cells to macrophages (Kallin et al., 2012). Collectively, these and other studies (Ficz et al., 2011; Williams et al., 2011b; Wu et al., 2011) implicate Tet1 and Tet2 in balancing stem self-renewal and differentiation and in maintaining cellular identity. Our results suggest that the Tet1 and Tet2 proteins participate in cell-fusion-based remodeling of the somatic genome induced by EGCs. We show that Tet1 is important for initiating imprint erasure in this model system and provide evidence that this is achieved through a combination of DNA replication-dependent and -independent steps.

## Experimental Procedures

### Cell Culture and Fusion

Epstein-Barr-virus-transformed human B clones (hB), abelson-transformed mouse B^*Oct4*-*GFP*^ cells (mB), and mouse B^*Oct4*-*GFP*/*Peg1*-*βgal*^ cells (2RB) were cultured as described before (Pereira et al., 2008). Mouse EGCs (Tada et al., 1997), ESCs (ESL21), female (Pgk12) ESCs (Zvetkova et al., 2005), and mutant ESCs lacking Dnmt1 (*Dnmt1*^−/−^) (Li et al., 1992) or Dnmt3a and Dnmt3b (*Dnmt3a*/*b*^*−*/*−*^) (Chen et al., 2003) were cultured as previously described (Pereira et al., 2010). EGCs or ESCs were fused with either human B-lymphocytes, mB, or 2RmB in a 1:1 ratio with the use of 50% polyethylene glycol (pH 7.4) (PEG 1500; Roche Diagnostics, Mannheim, Germany) as described previously (Pereira et al., 2010) and detailed in the Supplemental Experimental Procedures.

### Bisulfite Sequencing Analysis

Bisulfite modification of DNA was carried out with the EZ DNA Methylation Kit (ZymoGenetics, Washington, USA) according to the manufacturer’s recommendations. PCR primers that specifically recognize bisulfite-converted mouse or human DNA are listed in the Supplemental Experimental Procedures.

### 5hmC Detection

Genomic DNA (10μg) was treated with (+T4) or without (−T4) T4 Phage β-glucosyltransferase (T4-BGT; New England Biolabs, Massachusetts, USA) according to the manufacturer’s instructions and evaluated as detailed in the Supplemental Experimental Procedures.

### HpaII Resistance Assay

Genomic DNA (10μg) was digested with 50 U of HpaII (NEB), 100 U of MspI (NEB), or no enzyme (mock digestion) at 37°C for 4 hr, followed by Proteinase K treatment for 30 min at 40°C. The HpaII-resistant fraction was quantified by qPCR with the use of primers designed around at least one HpaII site, then normalized to a region lacking these sites (listed in the Supplemental Experimental Procedures) and the mock digestion control.

### Quantitative RT-PCR Analysis

RNA extraction was performed as described previously (Pereira et al., 2010). Mouse- and human-specific primers are listed in the Supplemental Experimental Procedures.

### Tet1 and Tet2 Knockdown

Short hairpin RNA (shRNA) sequences were cloned into pSuper.neo+GFP vectors (Oligoengine, Washington, USA). We electroporated 15 μg of empty (control) or shRNA-containing vectors into 5 × 10^6^ mouse EGCs by Amaxa Nucleofector 2b (Lonza, Basel, Switzerland).

shTet1: tgtagaccatcactgttcgac (Williams et al., 2011b)
shTet2: gctctgaacagtattcaaagc (Ito et al., 2010)

GFP^+^ EGCs were sorted with fluorescence-activated cell sorting (FACS) 12 hr after electroporation and replated for 24 hr before being harvested and used for characterizations and fusion experiments.

### Western Blot Analysis

Western blot analysis was performed as previously described (Pereira et al., 2010) and is detailed in the Supplemental Experimental Procedures.

### Immunofluoresence, BrdU, and X-Gal Staining

BrdU incorporation and immunofluorescence detection was performed as described previously (Tsubouchi et al., 2013). X-gal staining was performed as detailed in the Supplemental Experimental Procedures.

## Supplemental Information

Document S1. Supplemental Experimental Procedures and Figures S1–S4
