# Supplementary material for: Different Roles for Tet1 and Tet2 Proteins in Reprogramming-Mediated Erasure of Imprints Induced by EGC Fusion
Source: Mol Cell. 2013 Mar 28;49(6):1023–33. doi: 10.1016/j.molcel.2013.01.032 (PMC3613797; doi:10.1016/j.molcel.2013.01.032)
Supplement: Document S1. Supplemental Experimental Procedures and Figures S1–S4 [file mmc1.pdf]

Molecular Cell, Volume *49*

Supplemental Information

Different Roles for Tet1 and Tet2 Proteins in Reprogramming-Mediated Erasure of Imprints Induced by EGC Fusion

Francesco M. Piccolo, Hakan Bagci, Karen E. Brown, David Landeira, Jorge Soza-Ried, Amelie Feytout, Dylan Mooijman, Petra Hajkova, Harry G. Leitch, Takashi Tada, Skirmantas Kriaucionis, Meelad M. Dawlaty, Rudolf Jaenisch, Matthias Merkenschlager, and Amanda G. Fisher

1. **Figure S1**. Reprogrammed Hybrids Generated with Mouse EGCs Show Reduced DNA Methylation of Imprinted Loci, Related to Figure 1

2. **Figure S2**. Reprogramming of a Silent Peg1 Imprinted Gene in Mouse B Cells following Fusion with EGs, Related to Figure 2

3. **Figure S3**. Characterization of Mouse EGC Lines, Related to Figure 3

4. **Figure S4**. Sensitivity of DNA Methylation to Tet Depletion, Related to Figure 5

5. **Supplemental Experimental Procedures**, Related to Experimental Procedures

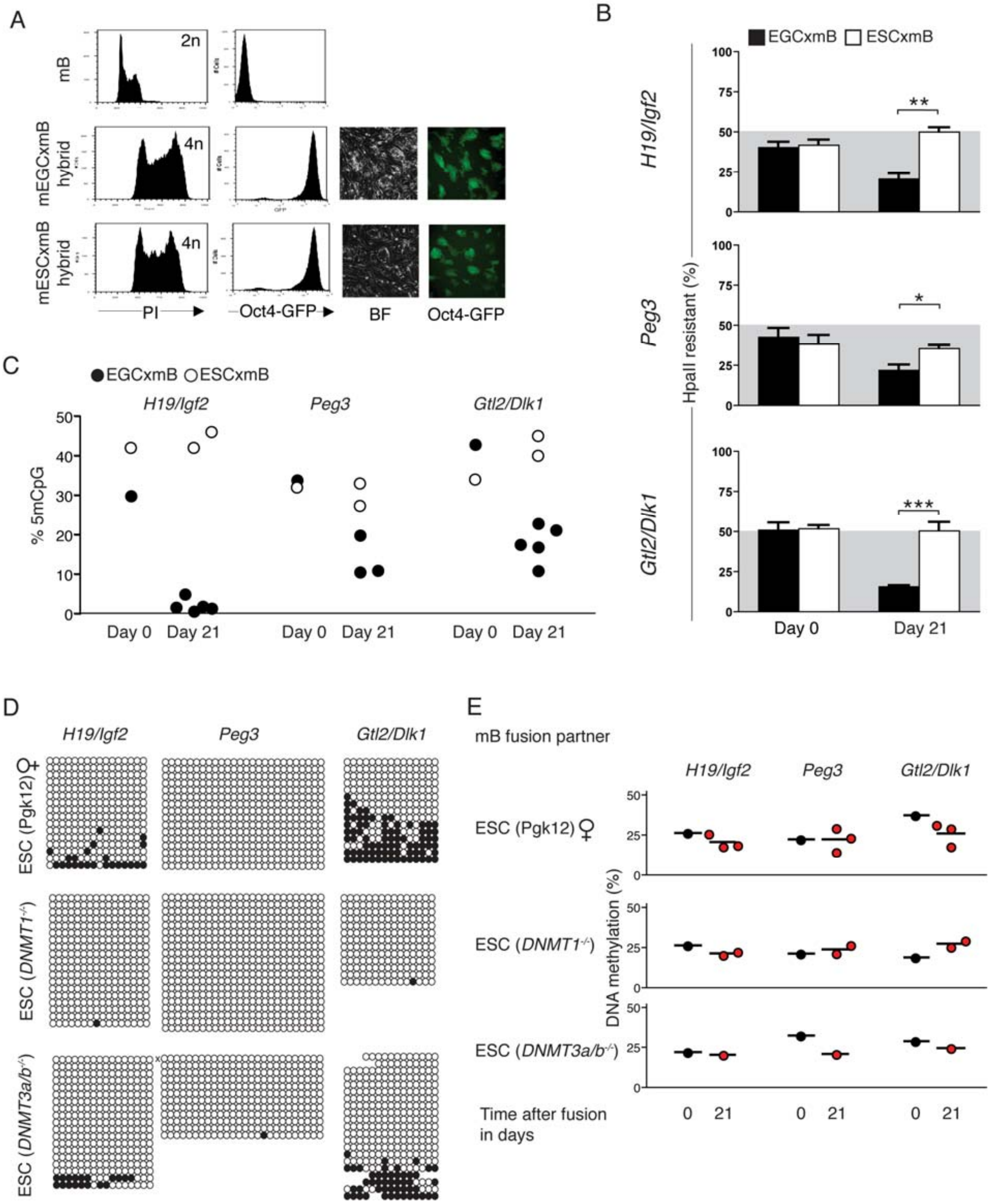

Figure S1. Reprogrammed Hybrids Generated with Mouse EGCs Show Reduced DNA Methylation of Imprinted Loci, Related to Figure 1

(A) DNA content (PI staining) and Oct4-GFP expression by mouse B$^{Oct4\text{-}GFP}$ cells (top panel, control mB) and representative hybrid clones isolated after fusing mB cells with EGCs

(middle panel) or ESCs (lower panel) are shown, where PI histograms indicate that hybrids are tetraploid (4n) and re-express Oct4-GFP.

(B) HpaII digest analysis of mouse *H19/Igf2, Peg3* and *Gtl2/Dlk1* ICRs in EG x mB and ESC x mB samples isolated before (Day 0) and after hybrid formation (Day 21). Results indicate the mean and standard error of four EG x mB (black histograms) and two ESC x mB (white histograms) independently isolated hybrid clones in which values obtained after HpaII digestion were normalized to undigested controls, as described in Figure 4A(*II*). Anticipated 50% levels of HpaII resistance are marked in grey. Statistical significance is indicated as * p-value < 0.05, ** p-value < 0.005, *** p-value < 0.0005, calculated with student t-tests.

(C) CpG methylation at ICRs before (Day 0) and after fusion (Day 21) of mouse B cells with EGCs (black dots) or ESCs (white dots) was estimated by bisulfite sequencing. Results shown are the mean DNA methylation levels (percentage 5mC-containing CpG/total CpG) for individual hybrid clones.

(D) Bisulfite analysis of three ICRs in female ESCs (Pgk12) and mutant ESCs that lack either Dnmt1 ($Dnmt1^{-/-}$) or Dnmt3a and Dnmt3b ($Dnmt3a/b^{-/-}$) are shown. Open circles represent unmethylated CpG, closed circles represent methylated CpG.

(E) CpG methylation at ICRs before (Day0, black dots) and after fusion (Day 21, red dots) of mouse B cells with female ESCs (Pgk12), mutant ESCs lacking Dnmt1 ($Dnmt1^{-/-}$) or Dnmt3a and Dnmt3b ($Dnmt3a/b^{-/-}$), estimated by bisulfite sequencing. Results show the average DNA methylation levels estimated by bisulfite sequencing for individual hybrid clones (Pgk12 and $Dnmt1^{-/-}$ fusions), or bulk cultures ($Dnmt3a/b^{-/-}$) isolated 21 days after cell fusion, where black lines denote mean values.

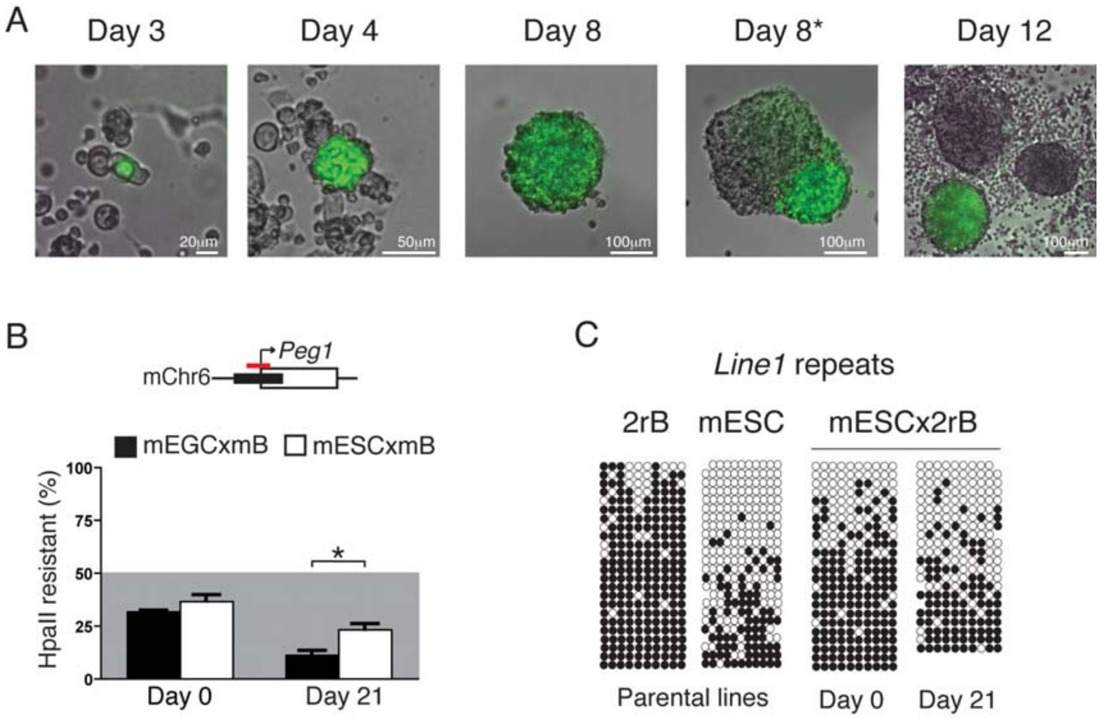

Figure S2. Reprogramming of a Silent Peg1 Imprinted Gene in Mouse B Cells Following Fusion with EGCs, Related to Figure 2

(A) Confocal images showing Oct4-GFP re-expression by a single EG x 2rB heterokaryon (at day 3) and by hybrid cells or colonies at 4, 8 and 12 days after fusion. Representative hybrid clones are shown in which Oct4-GFP expression was detected in all cells (day 4, 8) or where Oct4-GFP expression was either sectored (day 8*) retained or lost (day 12). Size bars indicate magnification.

(B) HpaII digest analysis of mouse *Peg1* ICR in EG x mB and ESC x mB samples isolated before (Day 0) and after hybrid formation (Day 21). Results show the mean and standard error of four EG x mB (black) and two ESC x mB (white) independently isolated Oct4-GFP$^+$ hybrid clones, where values after HpaII digestion were normalized to undigested controls, as described in Figure 4A(*II*). Anticipated 50% levels of HpaII resistance are marked in grey. Statistical significance is indicated as * p value < 0.05, calculated with student t-test.

(C) Bisulfite genome sequence analysis of mouse *Line1* repeat elements in 2rB and mESCs before fusion (parental lines), at fusion (Day 0) and in Oct4-GFP$^+$ 2rB x mESC hybrids collected 21 days after fusion. Open circles represent unmethylated CpG, closed circles represent methylated CpG.

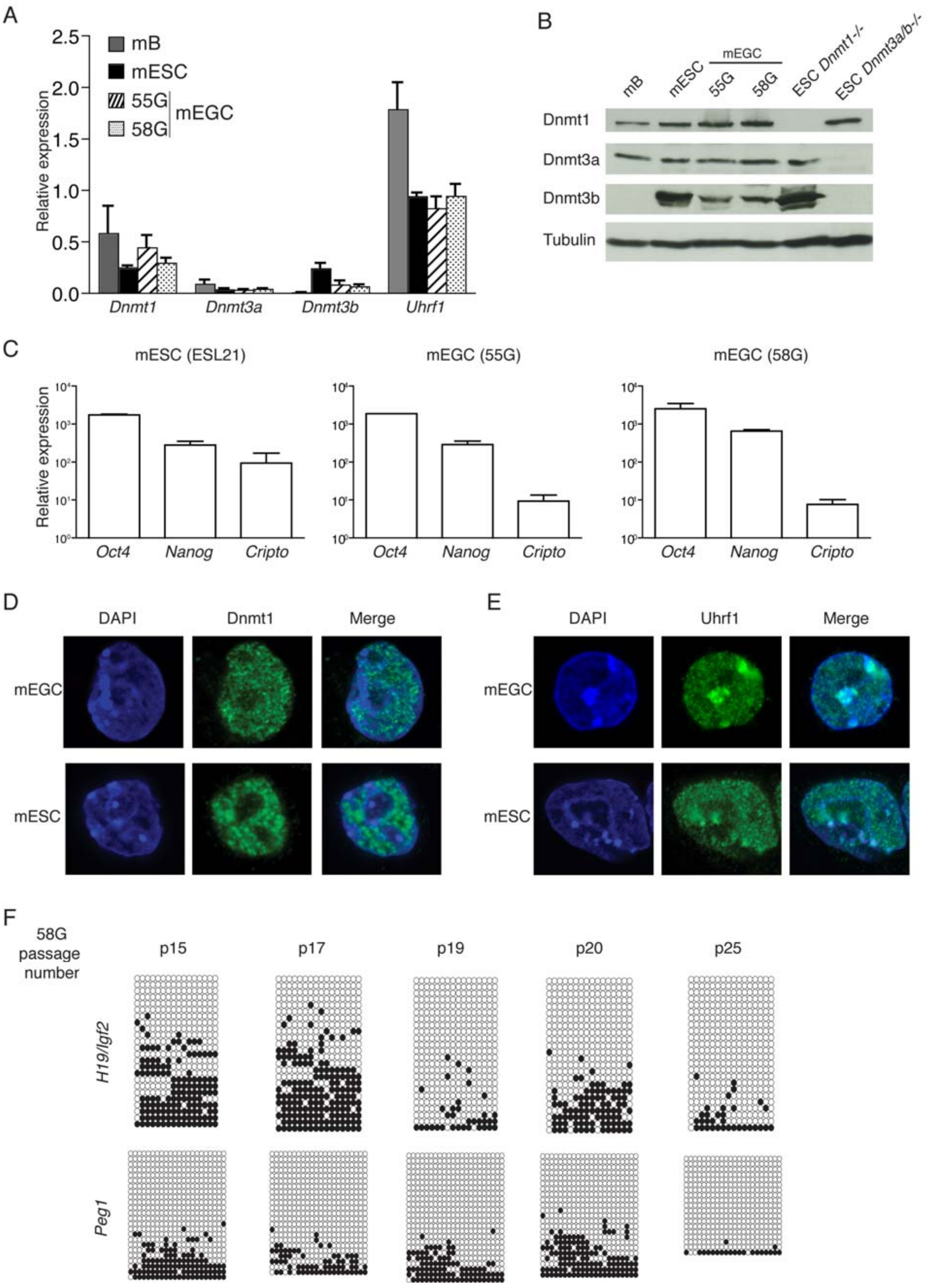

A
2.5
2.0
1.5
1.0
0.5
0.0
Relative expression
mB
mESC
55G
58G
mEGC
Dnmt1
Dnmt3a
Dnmt3b
Uhrf1
B
mB
mESC
mEGC
55G
58G
ESC Dnmt1-/-
ESC Dnmt3a/b-/-
Dnmt1
Dnmt3a
Dnmt3b
Tubulin
C
mESC (ESL21)
mEGC (55G)
mEGC (58G)
Relative expression
Oct4
Nanog
Cripto
D
DAPI
Dnmt1
Merge
mEGC
mESC
E
DAPI
Uhrf1
Merge
mEGC
mESC
F
58G passage number
p15
p17
p19
p20
p25
H19/Igf2
Peg1

Figure S3. Characterization of Mouse EGC Lines, Related to Figure 3

(A) qRT-PCR analysis of mouse *Dnmt1, Dnmt3a, Dnmt3b* and *Uhrf1* in mB, ESC (ESL21) and EGC lines (55G and 58G). Gene expression values were normalized to mouse *UBC* and indicate the mean and SD of two independent experiments.

(B) Western blots of Dnmt expression detected in whole cell extracts of mB, mESC, mEG (55G and 58G), ESCs (*Dnmt1*$^{-/-}$) and ESCa (*Dnmt3a/b*$^{-/-}$). Tubulin is shown as a loading control.

(C) Expression of pluripotency associated genes (*mOct4, mNanog* and *mCripto*) by mESC (ESL21) and mEGC lines (55G and 58G) was estimated by qRT-PCR and normalized to *UBC*. Values shown are the mean and SD of three independent experiments.

(D) Representative confocal images of mouse EGCs (upper) and ESCs (lower) labeled with anti Dnmt1 antibody (green).

(E) Representative confocal images of mouse EGCs (upper) and ESCs (lower) labeled with anti Uhrf1 antibody (green). DAPI counterstaining is shown in blue.

(F) Genome bisulfite sequencing analysis of mouse *H19/Igf2* and *Peg1* ICRs in 58G EGCs at sequential passages. Open circles represent unmethylated CpG, while closed circles represent methylated CpG.

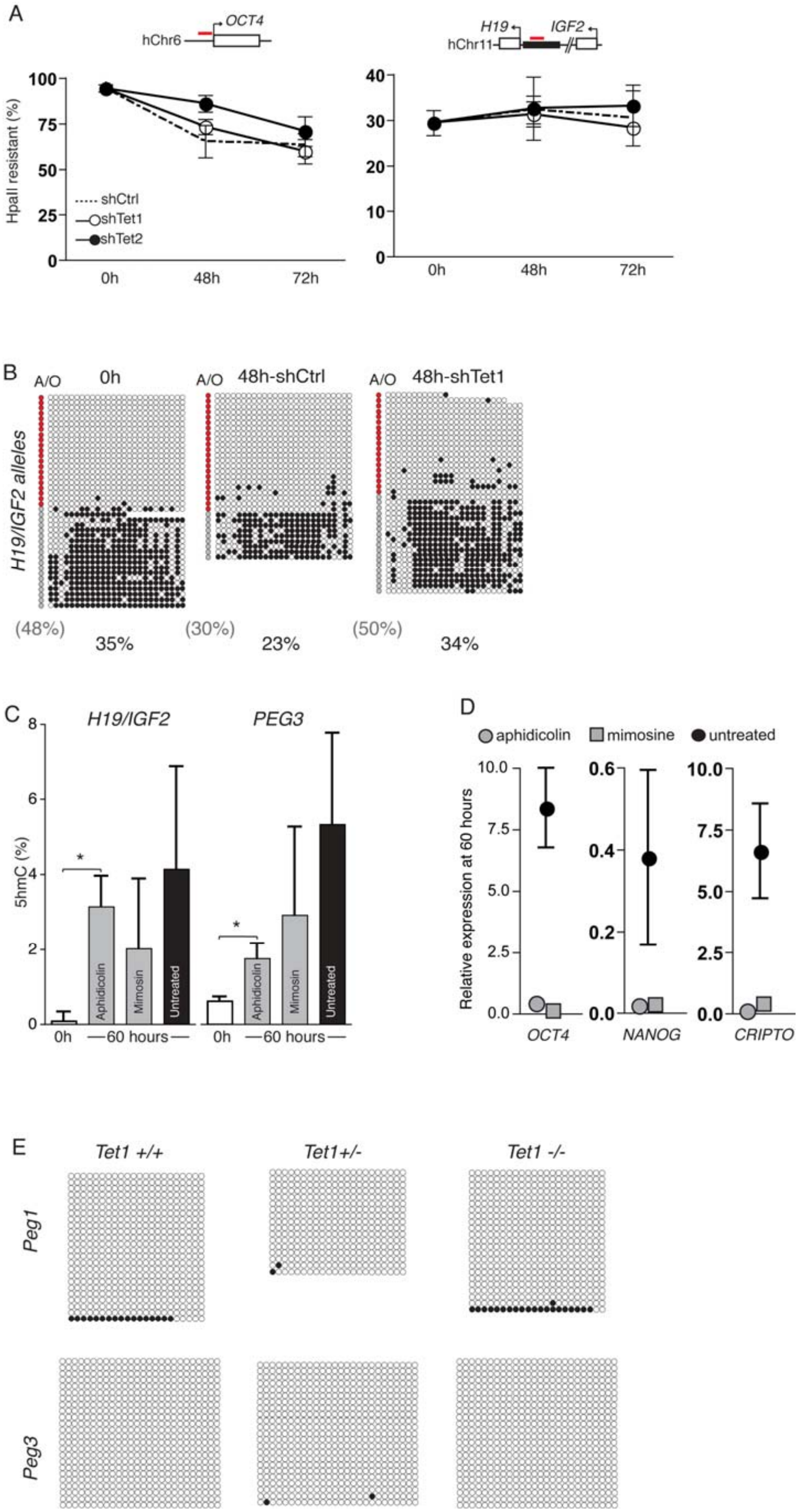

A
OCT4
hChr6
H19
IGF2
hChr11
HpaII resistant (%)
100
75
50
25
0
shCtrl
shTet1
shTet2
0h
48h
72h
40
30
20
10
0
B
A/O
0h
48h-shCtrl
48h-shTet1
H19/IGF2 alleles
(48%)
35%
(30%)
23%
(50%)
34%
C
H19/IGF2
PEG3
5hmC (%)
8
6
4
2
0
*
Aphidicolin
Mimosin
Untreated
0h
60 hours
D
aphidicolin
mimosine
untreated
Relative expression at 60 hours
10.0
7.5
5.0
2.5
0.0
0.6
0.4
0.2
OCT4
NANOG
CRIPTO
E
Tet1 +/+
Tet1+/-
Tet1 -/-
Peg1
Peg3

Figure S4. Sensitivity of DNA Methylation to Tet Depletion, Related to Figure 5

(A) HpaII digestion analysis at human *OCT4* promoter (left) and *H19/IGF2* ICR (right) in hB lymphocyte before (0h), and 48 and 72 hr after fusion with EGCs that had been transfected with empty plasmid (shCtrl, dashed line), shTet1 (open circles) or shTet2 (closed circles). Values shown are the mean and standard error of 3 independent experiments. Red bars mark the position of primer-amplified PCR products.

(B) Bisulfite analysis of the human *H19/IGF2* ICR in human B cells before (0h) and 48 hr after fusion with EGCs transfected with empty plasmid (shCtrl) or (shTet1). Allelic origin (A/O) is denoted in red (maternal) or grey (paternal) based on SNP, where frequency of paternal-derived allelic sequences in each condition is reported as percentage in brackets. Open circles represent unmethylated CpG and closed circles represent methylated CpG.

(C) 5hmC accumulation at human *H19/IGF2* and *PEG3* ICRs in heterokaryons formed between mEG and human B cells and either cultured with aphidicolin (2mg) or mimosine (350mM) for 60 hr or left untreated (grey and black histograms respectively). 5hmC levels were estimated as depicted in Figure 4A (*I*). Values indicate the mean and standard errors of three independent experiments where * shows p-value< 0.05, calculated with a student t-test.

(D) qRT-PCR analysis of human *OCT4, NANOG* and *CRIPTO* in heterokaryons formed between mEG and human B cells and cultured with aphidicolin (2mg) or mimosine (350mM) for 60 hr (grey circles and grey squares respectively) or left untreated (black circles). Gene expression values were normalized to *GADPH* and are shown as mean and SD of three independent experiments.

(E) Bisulfite analysis of *Peg1* and *Peg3* ICRs in genomic DNA samples isolated from keep sperm of wild type (+/+), heterozygote (+/-) and *Tet1*-null mice (-/-). Open circles represent unmethylated CpG, while closed circles represent methylated CpG.

Supplemental Experimental Procedures

Cell Fusion

EG/ES and hB/mB/2BR were mixed in a 1:1 ratio, washed and fused using PEG (50%, at 37°C over 1 min before dilution). Cells were washed and cultured in ES media at $0.5x10^6$ cells/cm^2. Where appropriate, to eliminate unfused human B cells, Ouabain (10^{-5} M; Sigma) was added to the medium 4 hr after cell fusion. Non-fused mouse EG or ESCs were eliminated by the addition of puromycin (1.5 μg/ml puromycin, Sigma) added from 6 to 12 hr after fusion. Reprogrammed hybrid (EG/ES x mB/2BR) clones were identified on the basis of Oct4-GFP expression, isolated using a pipette, dissociated with trypsin-EDTA, and then cultured in conditions used to maintain mouse EG/ESCs.

5hmC Detection

Glucosylated genomic DNA was digested with MspI (100U) or left untreated (mock digestion) at 37°C for 4 hr, followed by Proteinase K treatment for 30min at 40°C. The MspI-resistant fraction was quantified by qPCR using primers designed around at least one MspI site, and normalized to a region lacking MspI sites (see primers listed below) and to the mock digestion control. The percentage of 5hmC was calculated by subtracting the values obtained for T4 untreated MspI digestion from T4 pre-treated MspI digestion.

Western Blot Analysis

Western blot analysis was performed using appropriately diluted rabbit polyclonal anti-Dnmt1 (Novus Biological, NB100-264), chicken polyclonal anti-Dnmt3a (Abcam, ab1429), rabbit polyclonal anti-Dnmt3b (Abcam, ab16049-100), goat polyclonal anti-Oct3/4 (Santa Cruz Biotechnology, sc-8628), rabbit polyclonal anti-Nanog (Cosmo Bio, REC-RCAB0002P-F), rabbit polyclonal anti-Tet1 (Millipore, 09-872), rabbit polyclonal anti-Tet2 (Santa Cruz Biotechnology, sc-136926). As loading controls, blots were incubated with rabbit monoclonal anti-Tubulin (Sigma).

X-Gal Staining

Undifferentiated and differentiated hybrid clones were fixed for 15 minutes at room temperature in 0.1M phosphate buffer (pH 7.3), 5 mM EGTA, 2 mM MgCl2 , 0.2% glutareldahyde, washed in wash buffer (0.1M phosphate buffer (pH 7.3), 2 mM MgCl2) and incubated in staining buffer (0.1M phosphate buffer (pH 7.3), 2 mM MgCl2, 5mM potassium

ferrocyanide, 5mM potassium ferricyanide and 1 mg/ml X-gal) for 16 hr at 37ºC. Upon three final washes in wash buffer the stained plates were analysed using an inverted widefield microscope equipped with a 10X objective.

Lists of Primers Used in This Study

Bisufite Sequencing

Mouse Primers

Locus	Forward	Reverse
H19/Igf2 ICR	AAGGAGATTATGTTTTATTTTTGGA	AAAAAAACTCAATCAATTACAATCC
Peg3 ICR	TTGATAATAGTAGTTTGATTGGTAGGGTGT	ATCTACAACCTTATCAATTACCCTTAAAAA
DLK1/GTL2 ICR	GGAAGGAAAAGATAAAATGTAGAAA	CATAAATAAATAAACCCATAATCCC
Peg1/Mest ICR	GATTAGAGATTTATAAGGAAAGAG	CAACAAAAACAACAAACAACAAC
Line 1	GTTAGAGAATTTGATAGTTTTTGGAATAGG	TCAAACACTATATTACTTTAACAATTCCCA

Human Primers

ICR	Forward	Reverse
H19/Igf2	TGTTGAAGGTTGGGGAGATGGGA	CCCAAACCATAACACTAAAACCCTC

HpaII/MspI Digestion Primers

Mouse Primers

ICR	Digest./Normaliz.	Forward	Reverse
H19/Igf2	Digestion	CTGGATGCTCGTGTGAATGT	GCCTACAGTTCCCGAATCAC
	Normalization	ACAGCATTGCCATTTGTGAA	GACCATGCCCTATTCTTGGA
Dlk1/Gtl2	Digestion	CCTCGGGCAAAATATAGTGG	CGCAGCCTTCTCTGTGATCT
	Normalization	AAATGCAGAAAAGGGGGTGT	CAAAAGCCTTCAACACGTCA
Peg3	Digestion	CCCCAAACACCATCTGAACT	GTGCGTAGAGTGCTGTGCTC
	Normalization	TGGACATCTCTCCGCTTCTC	CAGAGGACCCTGACAAGGAG
Peg1/Mest	Digestion	AGGATGGGCGGGTTAGAG	AGGCAGCAAGCAGCAACT
	Normalization	AGGGGGTAGCGGGTCAATAC	AAGCAGAGAGGAGCAAGCAG

Human Primers

Locus	Digest./Normal	Forward	Reverse
H19/Igf2 ICR	Digestion	ACTGAAGCCCTCGGAGTGT	AGATCTTCAGGTCGGGCATT
	Normalization	GATAATGCCCGACCTGAAGA	GGGGTCATCTGGGAATAGGA
Peg3 ICR	Digestion	AAAACCCCTACAGGCAGGAC	GCGAAAATGCCCCTTCCT
	Normalization	GAAAACCCCTACAGGCAGGA	TTGTTTGCCGCAGTGGTG
SNRPN/SNRPN ICR	Digestion	ACTGCGGCAAACAAGCAC	CTCCTCAGACAGATGCGTCA
	Normalization	ACTGCGGCAAACAAGCAC	CAGGCTTCGCACACATCC
Oct4 Upstream TSS	Digestion	GTGTCTGTGGAAGGGGAAAA	AGTTTCTGTGGGGGACCTG
	Normalization	CCACTAGCCTTGACCTCTGG	CCACCATTAGGCAAACATCC
Oct4 Downstream TSS	Digestion	CTTGGAAGCTTAGCCAGGTC	CTCCAGGTGGTGGAGGTG
	Normalization	ATCACCTCCACCACCTGGA	GACACCTGGCTTCGGATTT

qRT-PCR Primers

Mouse Primers

Gene	Forward	Reverse
UBC	AGGAGGCTGATGAAGGAGCTTGA	TGGTTTGAATGGATACTCTGCTGGA
Oct4	CGTGGAGACTTTGCAGCCTG	GCTTGGCAAACTGTTCTAGCTCCT
Nanog	GAACTATTCTTGCTTACAAGGGTCTGC	GCATCTTCTGCTTCCTGGCAA
Cripto	CACCAACCCAGGGTATCAGTT	AGAGTTCTGTCCAGTGTCGTC
Dnmt1	AAGAATGGTGTTGTCTACCGAC	CATCCAGGTTGCTCCCCTTG
Dnmt3a	CGACCCATGCCAAGACTCACCTTCCAG	AGACTCTCCAGAGGCCTGGT
Dnmt3b	ACTGCCTGGAGTTCAGTAGGA	CCCTGTCTGATGGAGTTCGAC
Uhrf1	TGAAGCGGATGACAAGACTG	CAGGGCTCGTCCTCAGATAG
Tet1	GAGCCTGTTCCTCGATGTGG	CAAACCCACCTGAGGCTGTT
Tet2	TGTTGTTGTCAGGGTGAGAATC	TCTTGCTTCTGGCAAACTTACA
Bry	GCTCTCTCTCCCCTCCACACA	GCACTCCGAGGCTAGACCAGTT
Gata4	GAGGCTCAGCCGCAGTTGCAG	CGGCTAAAGAAGCCTAGTCCTTGCTT
Peg1/Mest	GGGTCGAGTATACGGTCCAA	TCGTCCTCTCCTTCTCCAAA

Human Primers

Gene	Forward	Reverse
GADPH	TCTGCTCCTCCTGTTCGACA	AAAAGCAGCCCTGGTGACC
OCT4	TCGAGAACCGAGTGAGAGGC	CACACTCGGACCACATCCTTC
NANOG	CCAACATCCTGAACCTCAGCTAC	GCCTTCTGCGTCACACCATT
CRIPTO	AGAAGTGTTCCCTGTGTAAATGCTG	CACGAGGTGCTCATCCATCA
